# Comment on Dao et al. Retrospective Analysis of Real-World Data for the Treatment of Obstructive Sleep Apnea with Slow Maxillary Expansion Using a Unique Expansion Dental Appliance (DNA). *Pathophysiology* 2023, *30*, 199–208

**DOI:** 10.3390/pathophysiology30040035

**Published:** 2023-10-07

**Authors:** G. Dave Singh

**Affiliations:** Division of Sleep Medicine, Stanford University, Stanford, CA 94305, USA; drsingh@drdavesingh.com

I found the recent article by Dao et al. titled “Retrospective analysis of real-world data for the treatment of obstructive sleep apnea with slow maxillary expansion using a unique expansion dental appliance (DNA)” [1] rather intriguing. The Abstract states that this is a “new technique for slow maxillary expansion”; however, the clinical effects of this device’s protocol on obstructive sleep apnea were first reported in 2011 [2] and published over the next 10 years [3,4]. Thus, although the authors conclude that their findings are “comparable to the findings in all previous studies”, I find this statement worrisome since the literature review of this particular article is deficient.

I believe this study has other errors. For example, in the Introduction, the authors state that “RME appliances … achieve expansion by applying centrifugal forces against the patient’s upper molars”. As a professor of orthodontics, I believe that such a clinical protocol would simply tip the teeth and produce a flawed measurement of the transpalatal width. In fact, orthodontists know there is no tendon in the human maxilla. In addition, in the Materials and Methods section, the authors state that “The DNA appliance comprises two upper and lower customized dental trays”, but the DNA appliance consists of only one, and the authors have mistakenly illustrated a similar device (the mRNA appliance) in Figure 1, which is also confirmed in a separate study by Katz et al. [5]. Moreover, the hotch-potch of treatment variables used in this particular study renders the findings untenable. 

I also believe this study did not report its findings objectively, which is exacerbated by poor data interpretation. For example, the authors claim the DNA appliance reduced the median AHI by 46% in their study. If so, these results do not meet the current criteria for medical success, since the AHI needs to be reduced by at least 50% on average with an AHI < 10. The authors also reported several non-significant correlations between changes in AHI and transpalatal width, airway volume, BMI, age, etc., and included findings that did not meet their power calculation for sample size. Moreover, the authors claim that their study has a “representation of all ethnicities” and “the data could be generalized to the US population”. However, only one African-American patient was included in the study, representing 1% of the sample population, compared to 13.5% of the US population, according to the US Census Bureau (2022) [6]. Therefore, bearing in mind the limitations of the study noted here, I believe the findings ought to be viewed with considerable caution.
